# Sulforaphane Protects the Male Reproductive System of Mice from Obesity-Induced Damage: Involvement of Oxidative Stress and Autophagy

**DOI:** 10.3390/ijerph16193759

**Published:** 2019-10-07

**Authors:** Li Huo, Yu Su, Gaoyang Xu, Lingling Zhai, Jian Zhao

**Affiliations:** 1 Department of Pharmacology, Shenyang Pharmaceutical University, Shenyang 110016, China; jerryhuo123@163.com (L.H.); suyu277@sina.com (Y.S.); xugaoyang520@sina.com (G.X.); 2 Department of Maternal and Child Health, School of Public Health, China Medical University, Shenyang 110001, China; llzhai@cmu.edu.cn

**Keywords:** sulforaphane, obesity, reproductive system, oxidative stress, autophagy

## Abstract

(1) *Background*: In recent decades, the prevalence of obesity has grown rapidly worldwide, thus causing many diseases, including male hypogonadism. Sulforaphane (SFN), an isothiocyanate compound, has been reported to protect the reproductive system. This research investigated the protective effect of SFN against obesity-induced impairment in the male reproductive system and explored the potential mechanism involved in mice. (2) *Methods*: One hundred thirty mice were divided into 5 groups (Control, DIO (diet-induced obesity), DIO + SFN 5 mg/kg, DIO + SFN 10 mg/kg, and DIO + SFN 20 mg/kg). The effects of SFN on the male reproductive system were determined based on the sperm count and motility, relative testes and epididymis weights, hormone levels, and pathological analyses. Oxidative stress was determined by measuring malondialdehyde (MDA), total antioxidant capacity (T-AOC), superoxide dismutase (SOD), glutathione (GSH), H_2_O_2_, catalase (CAT), and glutathione peroxidase (GSH-PX) levels. Protein expression of nuclear factor erythroid-2 related factor 2 (Nrf2), Kelch-like ECH-associated protein-1 (Keap1), Microtubule-associated protein light chain 3 (LC3), Beclin1, and P62 were determined by western blotting. (3) *Results*: High-fat diet (HFD)-induced obesity significantly decreased relative testes and epididymis weights, sperm count and motility, and testosterone levels but increased leptin and estradiol levels. SFN supplementation ameliorated these effects. Additionally, SFN administration inhibited the obesity-induced MDA accumulation and increased the SOD level. Western blot indicated that SFN had an important role in the downregulation of Keap1. Moreover, SFN treatment attenuated obesity-induced autophagy, as detected by LC3 and Beclin1. (4) *Conclusions*: SFN ameliorated the reproductive toxicity associated with obesity by inhibiting oxidative stress mediated by the nuclear factor erythroid-2 related factor 2/ antioxidant response element (Nrf2/ARE) signaling pathway and recovery of normal autophagy.

## 1. Introduction

Obesity, which is a metabolic disease, is generally considered to be associated with heredity, behavior, environment, and so on. The prevalence of obesity is continuing to increase at an alarming rate and has nearly tripled in the last 40 years [1]. In 2016, the prevalence of obesity among adults was 13% and the morbidity of overweight adults was 39% worldwide. The overweight and obesity rates among children and adolescents rose from 4% in 1975 to 18% in 2016 [2].

Studies have shown that obesity can affect paternal reproduction [3]. According to clinical experiments, the sperm count or motility are decreased and sperm DNA is damaged in men who are obese or overweight compared to men with normal BMI [4,5]. Studies have also shown that secondary (hypogonadotropic) hypogonadism (SH) in obese men will develop even without organic damage at the hypothalamic-pituitary axis [6,7]. The association between obesity and male SH are complex. It has recently been reported that there is a causal relationship between obesity and serum testosterone in men [8]. There is evidence showing that testosterone is indispensable for development of testis during puberty and maintenance of spermatogenesis in adults. The junction between Sertoli cells and cohesion between Sertoli and spermatogenic cells depends on testosterone. Reduced testosterone may contribute to SH in obese males [9]. Leptin derived from adipocytes is a key factor in regulating energy balance, the immune system, and reproduction. It has been shown that leptin regulates male reproduction by stimulating the hypothalamic-pituitary-gonadal (HPG) axis and that its level is significantly higher in obese males compared to normal weight males [10]. We have shown that a high leptin level contributes to decreasing the content of testosterone in the testis of obese mice [11].

Obesity has an adverse effect on male reproduction. Compared with normal people, the sperm count and motility are decreased in obesity man [4,5]. Our prior study also showed that obesity was related to male infertility [11]. Some studies and our prior study considered that oxidative stress may play the most important role [11,12] and that oxidative stress induced by obesity damages male reproduction maybe through superoxide generation, oxidative phosphorylation, and so on [13,14,15]. Therefore, an antioxidant substance may provide a protective effect on the damage induced by obesity.

Autophagy (programmed cell death type II), which is evolutionarily highly conserved, has a very important role in the maintenance of intracellular homeostasis by forming autophagosomes which then fuse with the lysosomal membrane to degrade the cytosolic components by hydrolytic enzymes [16]. Autophagy has an important effect on heat-induced spermatogenesis damage [17]. Knockout of autophagy-related gene 7 (Atg7) in murine germ cells results in male infertility [18]. Mice raised by a high-fat diet (HFD) have increased formation of autophagosomes [19]. HFD-induced spermatogenesis deficiency is mediated by autophagy and ameliorated by inhibition of the autophagic process [20].

**Figure 1:**
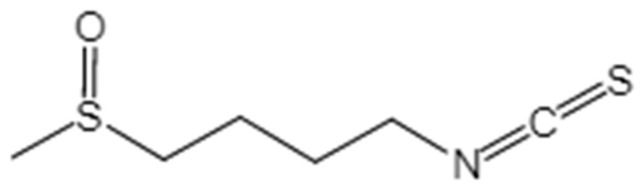
The chemical structure of sulforaphane (SFN).

Sulforaphane (SFN) (Figure 1) is an edible isothiocyanate that was generated in cruciferous plants, for example, cabbage, kale, cauliflower, and broccoli. There is evidence supporting the notion that SFNs can protect reproductive systems from the damage induced by di-N-butylphthalate (DBP) in pubertal mice [21]. Yang et al. [22] reported that SFN intervention can prevent the damage induced by cadmium in the reproduction of mice through the inhibition of oxidative stress mediated by the Nrf2/ARE signaling pathway. However, it is unknown whether SFN can protect mice from the injury to male reproduction caused by obesity. Thus, we determined the effects of SFN on the injury to male reproduction caused by obesity and evaluated the possible underlying mechanism.

## 2. Materials and Methods

### 2.1. Materials

SFN was purchased from the Toronto Research Chemicals (Toronto, Canada). Antibodies against Nrf2, Keap1, and LC3 were obtained from Abcam (London, Britain), and antibodies against Beclin1 and P62 were purchased from Cell Signaling Technology (Danvers, MA, USA).

### 2.2. Animals

C57BL/6J mice (Male, 4 or 5 weeks old) were obtained from Experimental Animal Center, Shenyang Pharmaceutical University, Shenyang, China. Animal use has been approved by Animal Use and Care Committee at Shenyang Pharmaceutical University (SYPU-IACUC-C2017-10-26-101). Mice were acclimated in the new environment at least one week before the beginning of this research. The mice were housed in controlled conditions of temperatures from 22 °C to 26 °C, 12 h light/dark cycle, and relative humidity of 40–60% and approached diet and water freely. All experiments and surgical procedures were carried out in accordance with the National Institute of Health Guide for the Care and Use of Laboratory Animals. All efforts were made to minimize animal suffering and to reduce the number of specimens used.

### 2.3. Diet

The control diet (3.85 kcal/g, *n* = 10) consisted of 10% fat-originated calories, 20% of protein-originated calories, and 70% of carbohydrate-originated calories. The HFD (*n* = 120) containing 45% kcal from fat was constituted of 73% standard chow diet, 20% lard, and 7% casein (Aoboxing Biotech Company Ltd., Beijing, China) and multiple trace vitamins [23]. Body weight of all mice was measured once a week.

### 2.4. Diet-Induced Obesity (DIO) Definition

As Levin et al. [24] designated, 40 mice with body weight gain (BWG) in the upper tertile after 8 weeks of an HFD were defined as DIO mice. The remaining mice were excluded from the experiment.

### 2.5. SFN Exposure

After being fed an HFD for 8 weeks, 30 mice were treated with different doses of SFN (5, 10, and 20 mg/kg) by gavage daily for 6 weeks.

### 2.6. Grouping

The mice were separated into 5 groups (10 mice per group) in this experiment, as below: Control, CON diet for 14 weeks; DIO, an HFD for 14 weeks; DIO + SFN 5 mg/kg group, an HFD for 14 weeks and SFN (5 mg/kg) by gavage daily from 8–14 weeks; DIO + SFN 10 mg/kg, an HFD for 14 weeks and SFN (10 mg/kg) by gavage daily from 8–14 weeks; and DIO + SFN 20 mg/kg group, an HFD for 14 weeks and SFN (20 mg/kg) by gavage daily from 8–14 weeks.

### 2.7. Experimental Procedure

**Figure 2:**
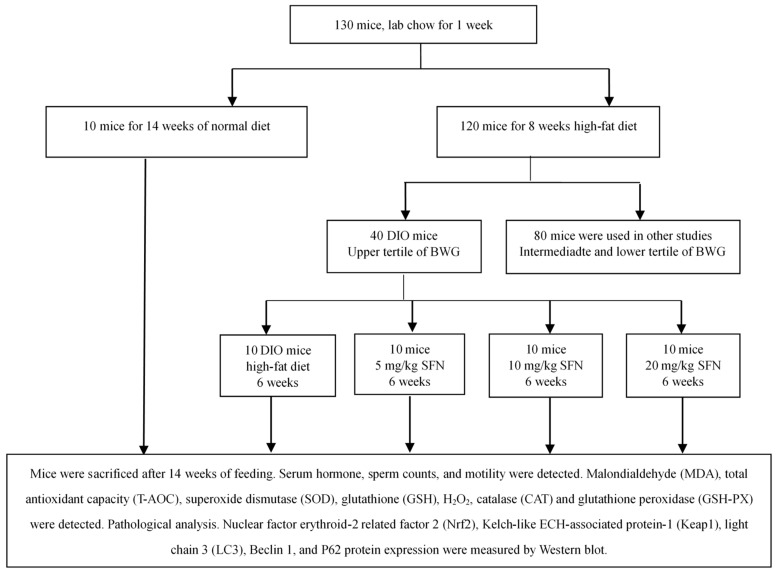
The flow diagram of experimental progress.

As depicted in Figure 2, 130 mice were acclimated for one week before the experiment and divided into two groups, as follows: ten mice were given a CON diet for 14 weeks, and the remaining 120 mice were given an HFD for eight weeks. Eight weeks later, 40 mice with BWG in the first tertile were designated as the DIO mice group. Forty DIO mice were further divided into four groups; the DIO mice were given an HFD for 6 weeks, and the other three groups were treated with both an HFD and SFN (5, 10, and 20 mg/kg; Toronto Research Chemicals, Toronto, Canada) for 6 weeks. All mice were sacrificed with ether 24 h at the end of this research.

### 2.8. Tissue Processing

After six weeks of administration, mice were euthanized with ether. After supine reflexion of supine position disappeared, the abdominal cavity was cut open with surgical scissors along the midline of the abdomen. The fat around the blood vessels was gently removed with small tweezers, and the extra fat covered on the blood vessels was wiped with cotton balls. Then, blood was collected from the inferior vena cava with 1-mL syringes. Blood samples were centrifuged to get sera, which were used for measuring hormone levels, including testosterone, leptin, and estradiol. Retroperitoneal fat, epididymal fat, epididymides, testes, kidneys and livers were excised after blood samples were collected and weighed. Epididymides of each mouse were excised free from fat to determine the sperm characteristics. Left testes of mice were produced as 10% homogenate to measure MDA, T-AOC, SOD, GSH, H_2_O_2_, CAT, and GSH-Px levels. Right testes of five mice in each group were excised for western blotting study. The remaining right testes were used for histological parameters.

### 2.9. Epididymal Sperm Count and Motility

The epididymides were collected immediately after obtaining blood samples and placed in 37 °C preheated M2 buffer and cut into pieces. Sperm motility (%) [25] were determined on a hemocytometer under a light microscope (Olympus, Tokyo, Japan) by a technician blinded to the group after incubation in 37 °C water bath. Two hundred sperm counted for each mouse was classified as follows: progressive motile; nonprogressive motile; and immotile. Sperm count (10^6^/mL) was measured on a hemocytometer under a light microscope (Olympus, Tokyo, Japan) before the sperm suspension was pretreated in the 60–70 °C water bath for 15 min. More procedures can be consulted in our previous study [23].

### 2.10. Light Microscopy

Testes were excised from fat, instantly fixed in 4% paraformaldehyde, dehydrated in gradient ethanol, cleared in xylene, embedded in paraffin, sliced at 4-μm thickness by a rotary microtome (Leica, Buffajrove, Illinois, USA), and subsequently stained with hematoxylin-eosin (HE) staining. The sections were viewed using a microscope (Olympus, Tokyo, Japan) and photographed.

### 2.11. Transmission Electron Microscope

Small pieces of testicular tissue were fixed in 2.5% glutaraldehyde solubilized in 0.1 M phosphate buffer (pH 7.2), postfixed in 1% osmium tetroxide, dehydrated in a gradient ethanol, and embedded in Epon812. Ultrathin slices were stained through uranyl acetate, followed by lead citrate, then inspected on a H-600 microscope (Hitachi, Tokyo, Japan), and photographed.

### 2.12. Hormone Measurements

Leptin (Merck Millipore, Darmstadt, Germany), testosterone (Enzo Life Science Inc., Farmingdale, NY, USA), and estrogen (Cayman Chemical Company, Ann Arbor, MI, USA) were determined by immunoassay kits under the guidance of the manufacturers’ protocols.

### 2.13. Biochemical Parameters

MDA (S0131, nmol·mg^-1^ protein), T-AOC (S0116, U·mg^-1^ protein), SOD (S0101, U·mg^-1^ protein), GSH (S0073, mgGSH·g^-1^ protein), H_2_O_2_ (S0038, mmol·g^-1^ protein), CAT (S0051, U·mg^-1^ protein), and GSH-PX (S0056, U·mg^-1^ protein) were determined by immunoassay kits from Beyotime Biotechnology (Jiangsu, China) in strict compliance with the manufacturer’s protocols.

### 2.14. Protein Preparation from Testes

Mice testes were dispersed with homogenizers in Radio Immunoprecipitation Assay (RIPA) lysis buffer (100 μL) containing 1 mmol/L protease inhibitor on ice. After that, the testes lysates were centrifuged for the supernatant. The concentration of supernatant was determined by bicinchoninic acid method under the instruction of the manufacturers’ protocol.

### 2.15. Western Blotting

Testicular protein (50 μg per well) was separated by 12% SDS-PAGE gels; then, the protein was transferred using the polyvinylidene fluoride (PVDF) membrane. The membrane was blocked with 4% skim milk. Immunoblotting was performed with the first antibody then the secondary antibody (1:5000) (ZSGB-BIO, Beijing, China). First antibody: anti-Nrf2 (ab31163, 1:1500 dilution; Abcam, London, Britain); anti-keap1 (ab119403, 1:1500 dilution; Abcam); anti-LC3B (ab63817, 1:1000 dilution; Abcam); anti-SQSTM1/P62 (#23214, 1:1000 dilution; Cell Signaling Technology, Danvers, MA, country); and anti-Beclin1 (#3738, 1:1000 dilution; Cell Signaling Technology). Signals were detected by chemiluminescence (ECL, Thermo Fisher Scientific, Waltham, MA, USA). Each experiment was repeated at least three times.

### 2.16. Statistical Analyses

Statistical analyses were performed by Statistical Product and Service Solutions (SPSS) 21.0 (IBM, Armonk, NY, USA). Results were expressed as mean ± standard deviation (SD). The difference among the groups was analyzed by ANOVA, *p* values < 0.05 were considered significant.

## 3. Results

### 3.1. Body Weight

**Figure 3:**
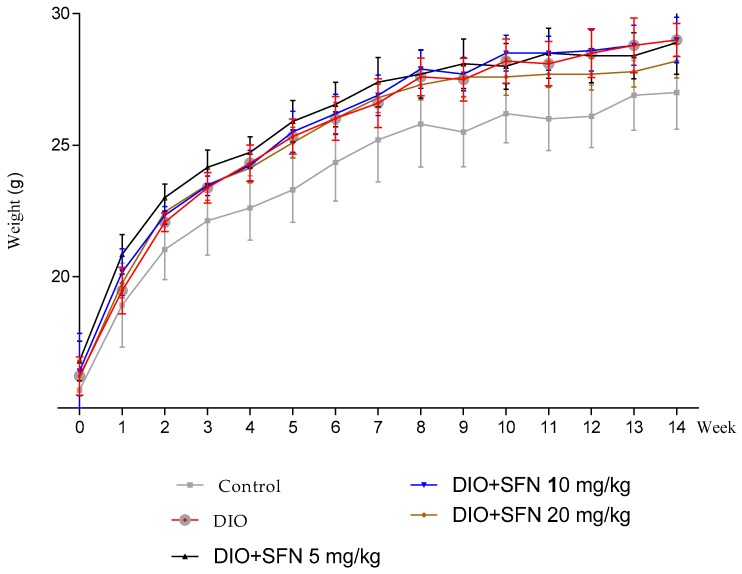
Effect of high-fat diet (HFD) on body weight changes in 14 weeks.

On the 8th weekend, body weight of the DIO (27.64 ± 0.72 g), DIO + SFN 5 mg/kg (27.69 ± 0.91 g), DIO + SFN 10 mg/kg (27.92 ± 0.73 g), and DIO + SFN 20 mg/kg groups (27.28 ± 0.57 g) were significantly higher compared to that of controls (25.75 ± 1.63 g) at 8 weeks (*p* < 0.01). At the end of 14 weeks, the body weight of DIO (28.95 ± 0.62 g), DIO + SFN 5 mg/kg (28.90 ± 1.20 g), DIO + SFN 10 mg/kg (28.95 ± 0.86 g), and DIO + SFN 20 mg/kg (28.21 ± 0.65 g) groups were higher than the body weight of controls (26.98 ± 1.40 g; *p* < 0.01; Figure 3). In our experiment, SFN treatment did not decrease the body weight of obese mice significantly.

### 3.2. Effects of SFN on Reproductive Organs Index and Sperm Characteristics in Obese Mice

The relative testes weight of the DIO group was significantly lower than the control mice (*p* < 0.01). The relative epididymis weight of the DIO mice was also lower than the control mice (*p* < 0.05). However, the relative epididymis weight of the obese mice was increased significantly after treatment of 10 or 20 mg/kg SFN (*p* < 0.05, Table 1).

The relative liver and kidney weights of the DIO mice were significantly lower than the control mice (*p* < 0.05). Compared with the DIO group, relative liver weight (DIO + SFN 5 mg/kg group) was significantly increased (*p* < 0.05), as with DIO + SFN 10,20 mg/kg mice (*p* < 0.01). The relative kidney weight of DIO + SFN 20 mg/kg mice was higher compared with DIO mice (*p* < 0.01, Table 1).

The relative epididymal fat and retroperitoneal fat weight of the DIO group was higher than control mice (*p* < 0.01). However, administration of 20 mg/kg SFN decreased the retroperitoneal fat weight of obese mice (*p* < 0.01, Table 1).

The sperm count and motility of DIO mice was also lower than the control group (*p* < 0.01). After supplementation of 5 mg/kg SFN (*p* < 0.05), 10 mg/kg SFN, and 20 mg/kg SFN (*p* < 0.01), the sperm count of the obese mice increased. In addition, 5, 10, and 20 mg/kg SFN also increased the sperm motility of the DIO mice (*p* < 0.01).

### 3.3. Effects of SFN on Testicular Histology in Obese Mice

**Figure 4:**
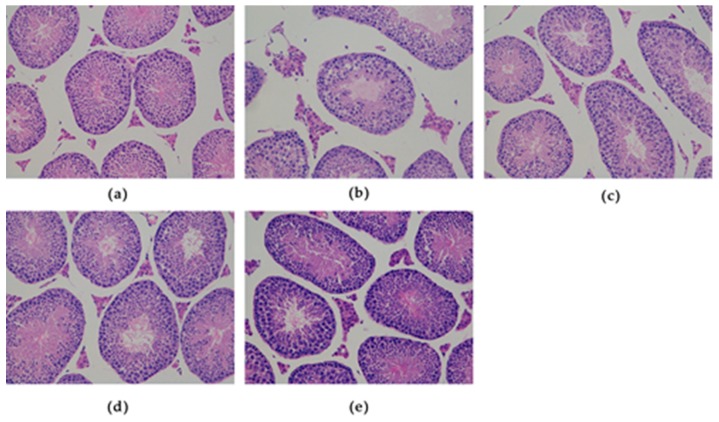
Effects of SFN on the morphology of testis in obese mice: Light microscopic images display the control group (**a**), DIO group (**b**), DIO + SFN 5 mg/kg group (**c**), DIO + SFN 10 mg/kg group (**d**), and DIO + SFN 20 mg/kg group (**e**). For all images: HE staining. Magnification ×40.

As showed in the Figure 4, morphological analyses of the testes in the DIO mice showed that the testicular structure was aberrant but that the abnormal structure was improved by exposure to SFN. Compared with the control mice (Figure 4a), adhesion of seminiferous epithelia which were atrophied was disrupted and the arrangement of seminiferous epithelia were sparse in the obese mice (Figure 4b). After the administration of SFN, the structure of seminiferous tubules returned to normal and seminiferous epithelia were arranged tightly (Figure 4c–e).

**Figure 5:**
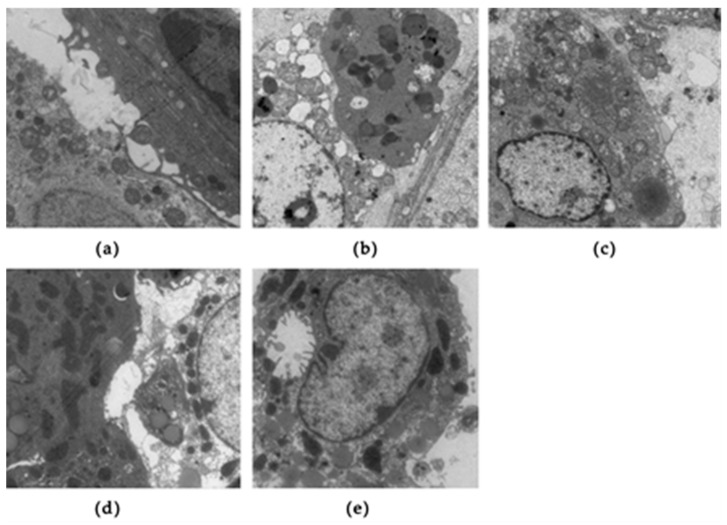
Effects of SFN on the morphology of testis in obese mice: Electron microscopy images display the control group (**a**), DIO group (**b**), DIO + SFN 5 mg/kg group (**c**), DIO + SFN 10 mg/kg group (**d**), and DIO + SFN 20 mg/kg group (**e**).

Electron microscopy of mouse testes was performed at week 14 (Figure 5). In the control mice (Figure 5a), the morphology of Leydig cells was normal with an abundance of organelles, such as smooth and rough endoplasmic and mitochondria, observed in Leydig cells. The Leydig cells had a small number of lysosomes and lipid droplets. In the DIO mice, the number of organelles were reduced and vacuolization was inspected in the Leydig cells. Mitochondria morphology was swollen and abnormal, and the number of lipid droplets increased (Figure 5b). SFN exposure improved these abnormalities. The number of organelles increased, while the number of lipid droplets and vacuoles decreased after administration of SFN (Figure 5c–e).

### 3.4. Effects of SFN on Serum Hormone Levels in Obese Mice

As shown in Table 2, the leptin levels of obese mice were significantly higher than control mice (*p* < 0.01). SFN (20 mg/kg) treatment decreased the leptin levels (*p* < 0.05). The testosterone levels were significantly lower than the control mice (*p* < 0.01). Administration of SFN increased the levels of testosterone (*p* < 0.05). DIO mice had increased fasting levels of estradiol than normal mice (*p* < 0.01). The supplementation of 10 mg/kg SFN (*p* < 0.05) and 20 mg/kg SFN (*p* < 0.01) decreased the estradiol levels.

### 3.5. Effects of SFN on Biochemical Parameters Related with Oxidative Stress in Obese Mice

As shown in Table 3, the CAT and T-AOC levels were not significantly different among the groups. The SOD (*p* < 0.01), GSH-PX (*p* < 0.05), and GSH (*p* < 0.01) levels of testicular tissues in obese mice were significantly lower than in the normal mice. However, only SOD levels of DIO + SFN groups (5–20 mg/kg) were significantly higher than control mice (*p* < 0.05). There was a significant increase in the H_2_O_2_ and MDA levels of the DIO group than control mice (*p* < 0.05). However, the supplementation of 20 mg/kg SFN decreased MDA levels (*p* < 0.01).

### 3.6. Effects of SFN on the Protein Expression in Obese Mice

**Figure 6:**
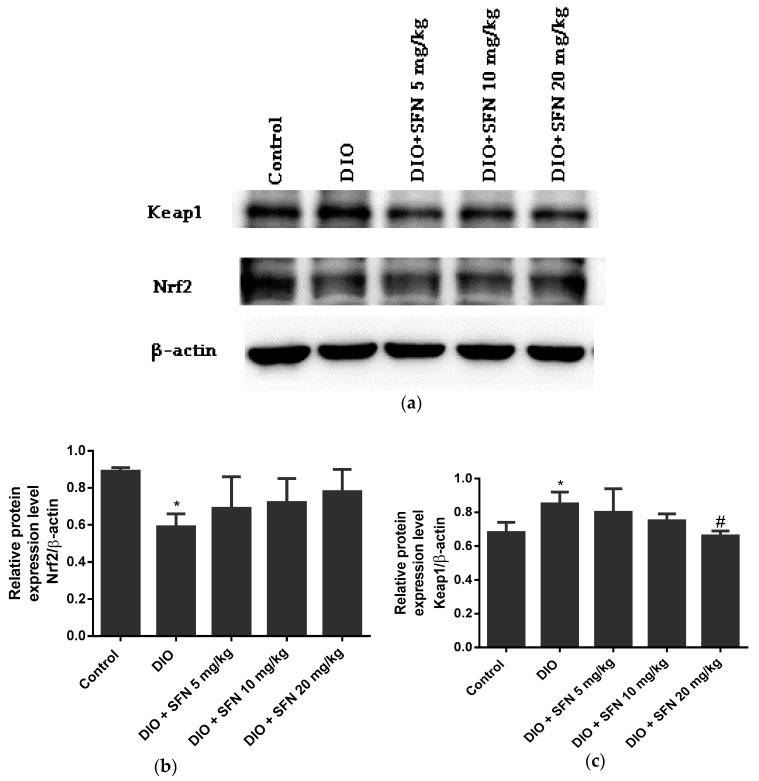
Effects of SFN on the protein expression related to oxidative stress in obese mice: (**a**) Protein expression of Nrf2 and Keap1. β-actin was used as a protein control to normalize the volume of protein expression: (**b**,**c**) Semiquantitative measurement of Nrf2 and Keap1. Data are expressed as mean ± Standard Error (SE). *n* = 3. * *p* < 0.05 vs. the control mice.

The Nrf2 levels of obese group were significantly lower than the control group (*p* < 0.05) (Figure 6b). The expression of Keap1 was significantly increased by obesity and decreased via treatment of 20 mg/kg SFN in obese mice (*p* < 0.05) (Figure 6c).

**Figure 7:**
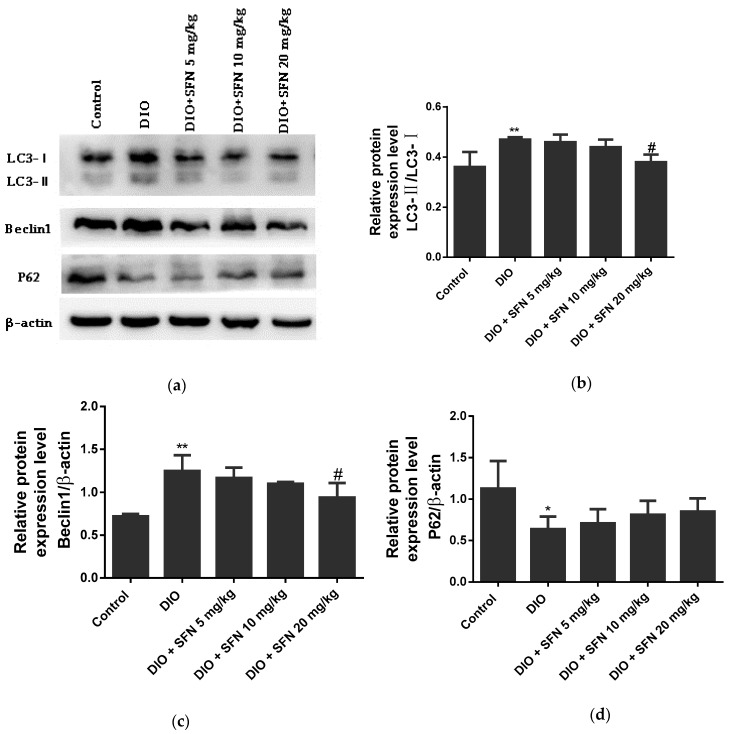
Effects of SFN on the protein expression related to autophagy in obese mice: (**a**) Protein expression of LC3, Beclin1, and P62. β-actin was used as a protein control to normalize the volume of protein expression: (**b**–**d**) Semiquantitative measurement of LC3, Beclin1, and P62. Data are expressed as mean ± SE. *n* = 3. * *p* < 0.05 and ** *p* < 0.01 vs. the control mice. ^#^ *p* < 0.05 vs. DIO mice.

In the DIO mice, the LC3-II/LC3-I (Figure 7b) and Beclin1 levels (Figure 7c) were significantly higher and the P62 levels (Figure 7d) were significantly lower than the normal mice. SFN blocked the significant increase in LC3-II/LC3-I and Beclin1 levels when co-administered with a high-fat diet.

## 4. Discussion

In our research, we inspected the protective effect of SFN against obesity-induced impairment on the male reproductive system and explored the potential mechanism underlying Nrf2/ARE-mediated oxidative stress and autophagy.

We found that the weights of DIO and the DIO + SFN groups were significantly higher compared to the control group at 8 weeks which meant that the model of obesity was successful. At 14 weeks, the weights of DIO mice were also significantly higher than the control mice, but there was no significant weight change after the treatment of SFN.

Our findings demonstrated that the male reproductive system was damaged after giving an HFD for 14 weeks, including a decrease of relative testes and epididymis weights and a decline of sperm count and motility. Erdemir et al. also found that the male reproductive system was damaged in obese mice [26]. SFN treatment enhanced relative testes, epididymis weights, and sperm count and motility. HE staining and electron microscopy also showed the protective effect of SFN on the pathological impairment to the testicular tissue induced by obesity. As shown by HE staining, the number of germ cells was decreased and arrangement of seminiferous tubules was sparse in the DIO group; however, this finding was ameliorated after SFN exposure. Electron microscopy showed that the organelles were reduced and that vacuolization was identified in the Leydig cells. The morphology of mitochondria was swollen and abnormal, and the number of lipid droplets was increased; however, SFN intervention improved these abnormalities. The number of organelles increased, while the number of lipid droplets and vacuoles decreased after administration of SFN. These results showed that SFN improved sperm quality of the DIO group and decreased testicular histopathological changes.

A number of studies have reported that reproductive hormonal profiles are changed in most obese males, such as increased estrogen and leptin levels and decreased testosterone levels [27,28,29]. We identified significantly higher serum estradiol and leptin levels and lower serum testosterone levels of the obese group than the control group. SFN administration enhanced the serum testosterone levels and decreased the serum estradiol and leptin levels. Testosterone has a crucial effect on testicular development, maintenance of secondary sexual characteristics, spermatogenesis, and the sex drive [30,31]. Studies have found that lower serum testosterone levels are responsible for obesity-induced male hypogonadism [32]. Leptin, a protein secreted from adipose tissue, has a critical effect on the regulation of the male reproductive system through modulating the HPG axis [33]. High leptin levels lead to the decrease of testosterone levels caused by obesity in murine [11]. SFN decreases the expression of leptin in adipose tissues and the serum/plasma concentration of leptin [21,34]. In the current experiment, we also found that SFN markedly reduced the serum leptin levels of obese mice and increased serum testosterone levels at the same time. Thus, SFN restored the imbalance of hormones induced by obesity.

Oxidative stress in testicular tissues of obese males is associated with male infertility and results from the increase in ROS, including superoxide anion, nitric oxide, and so forth, and from a decrease in antioxidant system activity. ROS damage nuclear and mitochondrial DNA and the plasma membranes of sperm and have a detrimental effect on the testicular environment [13]. We showed that oxidative stress of testicular tissues may contribute to the low testosterone levels of the DIO group [11]. In the present research, we measured oxidative stress markers, which included SOD, CAT, GSH-PX, T-AOC, GSH, H_2_O_2_, and MDA levels of testicular tissues. The MDA and H_2_O_2_ levels of the DIO group were higher than the control group. MDA levels, which are generally taken as an indicator of oxidative stress, of the DIO + SFN 20 mg/kg group were decreased compared to that of the DIO group. In addition to acting as independent signal molecules, H_2_O_2_ may be associated with the oxidative death cycle [35,36]. Although there was no statistical difference, the H_2_O_2_ levels of the DIO + SFN groups decreased than the DIO group. The SOD, GSH-PX, and GSH levels, which are important antioxidant enzymes in cells, of the DIO mice were significantly lower than the control mice. However, the SOD levels of the DIO + SFN groups were increased compared to the DIO mice. Yang et al. [22] reported that SFN treatment improved the activity of antioxidant enzymes, for example, SOD in the testicular tissues, which were exposed to cadmium and ameliorated the damage induced by oxidative stress. These results suggest that SFN enhances the antioxidative enzyme activity and protects the testes from the damage induced by oxidative stress.

Nrf2 is a key transcription factor encoded by the NFE3L2 gene and protects cells from damage induced by oxidative stress via regulation of αII antioxidant and detoxification enzymes, which have a critical effect on the maintenance of cell redox homeostasis [37]. In regular circumstances, Nrf2 is repressed via Keap1 and rapidly degraded via a ubiquitin-mediated proteasome in the cytoplasm [38,39]. Activation of Nrf2 in response to oxidative stress or activation of nucleophile results in displacement of Nrf2 from the cytoplasm to the nucleus. Nrf2 binding with ARE promoters stimulates transcription of antioxidative genes downstream and elevates the expression of antioxidant enzymes, which have a crucial effect on the maintenance of cell redox homeostasis [40]. We discovered that obesity increased the expression of Keap1 and decreased levels of Nrf2 as well as the activation of other antioxidant enzymes, indicating that obesity-induced oxidative stress improved by stimulation of the Nrf2 antioxidant pathway. SFN, an inducer of Nrf2, results in the stimulation of the Nrf2 antioxidant pathway and expression of downstream antioxidant enzymes by interacting with specific cysteine residues of Keap1 [40]. We found that SFN elevated the Nrf2 levels and reduced the Keap1 levels. In addition, SFN treatment decreased MDA and H_2_O_2_ levels and increased the antioxidant enzyme levels like SOD. The low Keap1 levels and high Nrf2 levels were associated with the high SOD expression. Consequently, SFN may protect the reproductive system from injury induced by oxidative stress via stimulation of the Nrf2/ARE signaling pathway.

Autophagy is activated under specific conditions, such as depletion of nutrition and growth factors, and under hypoxia [41]. In agreement with previous findings that mice fed an HFD upregulate the activation of autophagy [19], we found autophagy was stimulated in the obese mice. Beclin1 has a crucial effect on autophagy, including the formation of autophagosomes. LC3, which has two subtypes, including cytosolic protein LC3-I and membrane-bound protein LC3-II, is often used as a biomarker of the autophagy process. LC3-I in the cytoplasm converts into LC3-II, which is integrated into the membranes of autophagosomes [42]. P62 protein (sequestosome 1 (SQSTM1)) is a multifunctional scaffold protein that can recognize the ubiquitinated structures degraded by LC3-decorated autophagosomes [43]. In the present study, the LC3-II/LC3-I and Beclin1 levels in DIO mice were significantly higher and P62 levels were lower than the control mice, implying a role of autophagy in the testes of obese mice. Autophagy was associated with the survival and motility of sperm [44]. Spermatogenesis deficiency induced by an HFD is mediated by autophagy and ameliorated by inhibition of the autophagic process [20]. SFN treatment significantly inhibited the increased LC3-II/LC3-I and Beclin1 levels induced by obesity. Our study findings suggested that SFN protected the reproductive system from obesity-induced injury by restoration of normal autophagy. A previous study also indicated that SFN improved ischemia-induced detrusor overactivity by decreasing the enhancement of autophagy [45]. The finding that SFN improved testes impairment does not support the notion that inhibition of autophagy is a means of protecting the male reproductive system. Conversely, the activation of autophagy is indispensable in the process of decidualization of obese female mice induced by an HFD [20]. These researches possibly demonstrated that the roles of autophagy are different in pathophysiological processes. Consequently, further research should be conducted to elucidate the dual roles of autophagy.

This study clearly demonstrated that obesity induced by an HFD activated oxidative stress and autophagy and that the activation was associated with obesity-induced damage of the reproductive system. The interaction between oxidative stress and autophagy is not clear. A previous study showed that H_2_O_2_-activated autophagy is accompanied by increased expression of Beclin1 protein in human glioma cells [46]. N-2-mercaptopropionyl glycine (MTG), which is an antioxidant, inhibits autophagy of cardiac myocytes exposed to H_2_O_2_ in vivo and attenuates autophagic flux in cardiac myocytes of mice that are damaged by oxidative stress induced by ischemia/reperfusion [47]. In hypertension, oxidative stress can activate autophagic cell death, which can result in cardiac remodeling and dysfunction. Adiponectin, which has antioxidant potential, inhibits autophagy induced by oxidative stress via attenuating adenosine monophosphate-activated protein kinase/ mechanistic target of rapamycin (AMPK/mTOR) signaling induced by H_2_O_2_ [48]. Consequently, we hypothesize that SFN exerts cytoprotective actions on the testes of mice exposed to an HFD through inhibition of autophagy induced by oxidative stress via inhibition of Nrf2/ARE signaling.

## 5. Conclusions

In conclusion, this research confirmed that SFN ameliorates male reproductive damage related to obesity by inhibition of oxidative stress regulated by the Nrf2/ARE signaling pathway and recovery of normal autophagy. The potential protective effect of SFN on the damage induced by obesity maybe a way to treat reproductive system disease clinically in the future.

## Figures and Tables

**Table 1:** Effects of SFN on reproductive organs index and sperm characteristics in obese mice (X ± SD).

Group	*n*	Relative Tes.Weight (g/100g)	Relative Epididymis Weight (g/100)	Relative Sem Weight (g/100g)	Relative Liver Weight (g/100g)	Relative Kidney Weight (g/100g)	Relative Epi Fat (g/100g)	Relative Ret Fat (g/100g)	Sperm Motility (%)	Sperm Count (×10^6^/mL)
Control	10	0.84 ± 0.09	0.30 ± 0.03	0.95 ± 0.10	4.44 ± 0.24	1.21 ± 0.07	1.04 ± 0.21	0.19 ± 0.09	30.50 ± 2.22	4.91 ± 0.28
DIO	10	0.74 ± 0.04 **	0.28 ± 0.02 *	1.0 ± 0.11	4.18 ± 0.17 **	1.15 ± 0.06 *	1.42 ± 0.25 **	0.37 ± 0.08 **	21.50 ± 1.97 **	3.72 ± 0.28 **
DIO + SFN 5 mg/kg	10	0.74 ± 0.03	0.28 ± 0.03	0.98 ± 0.15	4.36± 0.17 ^#^	1.17 ± 0.03	1.48 ± 0.15	0.37 ± 0.09	23.80 ± 2.10 ^#^	4.04 ± 0.40 ^##^
DIO + SFN 10 mg/kg	10	0.76 ± 0.04	0.30 ± 0.04 ^#^	0.95 ± 0.13	4.43 ± 0.15 ^##^	1.18 ± 0.07	1.35 ± 0.18	0.35 ± 0.10	29.10 ± 1.61 ^##^	4.79 ± 0.16 ^##^
DIO + SFN 20 mg/kg	10	0.77 ± 0.04	0.31 ± 0.03 ^##^	0.97 ± 0.13	4.48 ± 0.20 ^##^	1.22 ± 0.07 ^##^	1.33 ± 0.11	0.24 ± 0.09 ^##^	31.20 ± 2.96 ^##^	4.76 ± 0.55 ^##^

Data are mean ± SD.* *p* < 0.05 and ** *p* < 0.01 vs. Control group; ^#^*p* < 0.05 and ^##^*p* < 0.01 vs. DIO group. Testis—Tes. Seminal vesicles—Sem. Retroperitoneal—Ret. Epididymal—Epi. Relative organs index = organ weight/ body weight × 100. Sperm motility = total motile sperm/all count sperm × 100.

**Table 2:** Effects of SFN on serum hormone levels in obese mice (X ± SD).

Group	*n*	Leptin (ng/mL)	Testosterone (pg/mL)	Estradiol (pg/mL)
Control	10	1.56 ± 0.37	4724.28 ± 619.50	14.15 ± 3.24
DIO	10	4.46 ± 1.35 **	3580.00 ± 808.21 **	19.10 ± 2.04 **
DIO + SFN 5 mg/kg	10	3.70 ± 1.41	4622.02 ± 864.37^#^	18.31 ± 3.43
DIO + SFN 10 mg/kg	10	3.62 ± 0.86	4651.58 ± 905.40^#^	16.10 ± 3.15^#^
DIO + SFN 20 mg/kg	10	3.49 ± 0.60^#^	4865.13 ± 519.91^##^	14.27 ± 3.00^##^

Data are mean ± SD. * *p* < 0.05 and ** *p* < 0.01 vs. Control group; ^#^ *p* < 0.05 and ^##^ *p* < 0.01 vs. DIO group.

**Table 3:** Effects of SFN on SOD, CAT, GSH-PX, T-AOC, GSH, H_2_O_2_, and MDA levels in obese mice (X ± SD).

Group	*n*	SOD (U/mg prot)	CAT (U/mg prot)	GSH-Px (U/mg prot)	T-AOC (U/mg prot)	GSH (mg/g prot)	H_2_O_2_ (mmol/gprot)	MDA (nmol/mg prot)
Control	10	234.65 ± 28.81	14.40 ± 1.69	57.27 ± 10.14	0.70 ± 0.21	146.23 ± 16.63	4.53 ± 0.72	1.48 ± 0.68
DIO	10	171.76 ± 40.66 **	13.70 ± 2.20	44.97 ± 10.71 *	0.59 ± 0.22	100.89 ± 16.34 **	18.13 ± 3.20 **	6.37 ± 1.11 **
DIO + SFN 5 mg/kg	10	234.51 ± 38.57 ^##^	13.90 ± 2.70	45.14 ± 8.03	0.61 ± 0.11	94.62 ± 12.30	16.11 ± 4.61	5.64 ± 0.91
DIO + SFN 10 mg/kg	10	249.96 ± 40.29 ^##^	15.75 ± 2.93	46.14 ± 9.01	0.61 ± 0.19	104.98 ± 20.15	16.57 ± 2.98	5.82 ± 1.35
DIO + SFN 20mg/kg	10	251.52 ± 45.25 ^##^	15.41 ± 2.85	46.48 ± 9.65	0.62 ± 0.18	112.63 ± 19.54	16.25 ± 2.72	4.65 ± 1.09 ^##^

Data are mean ± SD. * *p* < 0.05 and ** *p* < 0.01 vs. Control group; ^#^ *p* < 0.05 and ^##^ *p* < 0.01 vs. DIO group.
